# A Six-Year Surveillance of Nasal Methicillin-Resistant *Staphylococcus aureus* Colonization on Intensive Care Unit Admission: Do We Need Screening?

**DOI:** 10.3390/idr17060136

**Published:** 2025-10-24

**Authors:** Esma Eryilmaz Eren, Nursel Karagöz, Esma Saatçi, İlhami Çelik, Emine Alp Meşe

**Affiliations:** 1 Department of Infectious Diseases and Clinical Microbiology, Kayseri City Training and Research Hospital, University of Health Sciences, 38080 Kayseri, Türkiye; ilhamicelik@hotmail.com; 2 Infection Control Committee, Kayseri City Training and Research Hospital, 38080 Kayseri, Türkiye; 3 Department of Medical Microbiology, Kayseri City Training and Research Hospital, 38080 Kayseri, Türkiye; 4 Department of Infectious Diseases and Clinical Microbiology, Ankara Bilkent City Hospital, Yıldırım Beyazıt University, 06800 Ankara, Türkiye

**Keywords:** colonization, methicillin-resistant *Staphylococcus aureus*, screening

## Abstract

**Background:** Methicillin-resistant *Staphylococcus aureus* (MRSA) colonization is a risk factor for potential staphylococcal infection and outbreaks. Although it is recommended to obtain a swab culture to detect nasal colonization its necessity in low-prevalence countries is debated. The aim of this study was to determine the prevalence of MRSA nasal colonization, the rate of invasive infection development, and the risk factors for invasive infections in patients admitted to the intensive care unit. **Materials and Methods:** This retrospective study included patients who were followed up in one of the adult intensive care units at Kayseri City Training and Research Hospital between 1 January 2019 and 31 December 2024 (6 years) and from whom a culture was taken at the time of hospital admission to detect MRSA colonization in the nose. MRSA carriers were examined for the development of any invasive infection caused by MRSA within 28 days of their relevant admission. **Results:** Over a total period of six years, nasal swab samples were collected from 22,913 patients, and MRSA colonization was detected in 939 (4.0%). Of the patients with MRSA colonization, 32 (3.4%) were excluded from the analysis because they already had invasive MRSA infection. Additionally, 431 patients (45.8%) were excluded from the analysis because they were discharged or died within the first seven days of their admission. Consequently, invasive MRSA infection developed within 28 days in 29 of the 476 patients with MRSA colonization (6.0%). Patients who developed invasive infection had a higher rate of chronic renal failure (*p* < 0.001), hemodialysis (*p* < 0.001), central venous catheter (*p* = 0.028), staying in nursing home (*p* = 0.001), and a history of hospitalization within the last 90 days (*p* = 0.015). In the multivariable regression analysis, routine hemodialysis (OR: 5.216, *p* = 0.015), nursing home stay (OR: 3.668, *p* = 0.014), and a history of hospitalization within the last 90 days (OR: 2.458, *p* = 0.028) were found to be risk factors for developing invasive infection. The most common invasive infections were ventilator-associated pneumonia (*n* = 9), surgical site infection (*n* = 7), and catheter-related bloodstream infection (*n* = 6). All 29 strains were susceptible to vancomycin, linezolid, and daptomycin, while one strain was resistant to teicoplanin (3.5%). **Conclusions:** MRSA colonization has been detected in 4% of patients admitted to the intensive care unit. Screening should be performed because MRSA colonization may be a risk factor for invasive infections; however, screening all patients would be prohibitively expensive and labor-intensive. Instead, it may be more appropriate to identify risk factors and then screen select patients.

## 1. Introduction

*Staphylococcus aureus* is one of the most common causative agents of healthcare-associated infections recently [1]. Hospital infections caused by *S. aureus* have become an increasingly significant problem in recent years due to their multidrug resistance to methicillin and many other antibiotics [2]. Methicillin-Resistant *S. aureus* (MRSA) strains have become a serious global problem due to limited treatment options and high treatment costs [3]. In Türkiye, 12% of staphylococci isolated from an intensive care unit were MRSA [4]. However, according to the World Health Organization (WHO) report, MRSA rate in bloodstream infections in 2021 was over 30 percent [5]. Also, according to the Antimicrobial resistance surveillance in Europe report, the proportion of MRSA strains exceeded 30% in our country [6].

Colonization with *S. aureus* is the first step in a potential staphylococcal infection. In nasal carrier patients, these bacteria can colonize the skin and mucous membranes and spread to the patient from vascular access sites, causing infection [7]. In particular, it has been found that the incidence of *S. aureus* colonization and infection is significantly increased in patients with *S. aureus* nasal colonization in intensive care units. It has been reported that failure to take the necessary infection control measures after admitting a patient colonized with MRSA to the intensive care unit can lead to an outbreak [8].

To prevent possible outbreaks and invasive infections, it is recommended that swab cultures be taken to detect nasal colonization during hospital admission and that isolation be applied to patients carrying MRSA. In regions with low prevalence, this screening is not recommended as it is not cost-effective [9,10,11]. Although there are facilities in our country that perform MRSA screening, the prevalence rate among hospitalised patients, the rate of invasive infections caused by MRSA in patients defined as carriers, and the frequency of infection development from colonisation are unknown.

The aim of this study is to determine the prevalence of nasal MRSA colonization among patients admitted to the adult intensive care units of our hospital and to identify risk factors for the development of infection from MRSA colonization.

## 2. Materials and Methods

### 2.1. Study Design and Patients

This retrospective study was conducted in a tertiary care hospital with 175 adult intensive care beds and a total bed capacity of 1600.

Patients treated in the adult intensive care unit of Kayseri City Hospital between 1 January 2019 and 31 December 2024 (6 years) and tested for MRSA colonization with a nasal culture on admission were included.

Patient data were obtained from the records of the Infection Control Committee. Among these patients, those identified as MRSA carriers were evaluated for the development of any invasive MRSA-related infection within 28 days following the corresponding hospitalization. Those who developed MRSA infection were classified as “group 1,” while patients who did not develop invasive infection were classified as “group 2.” Invasive infections include all hospital-acquired infections, such as catheter-related or primary bloodstream infections, pneumonia or ventilator-associated pneumonia, and upper urinary tract infections.

In order to identify risk factors for the development of invasive infections in patients with nasal MRSA colonisation, a comparison was made between those with invasive infections and those without. Variables such as age, gender, comorbidities, concomitant infections and invasive procedures were analysed to determine their relationship with the development of infection.

Patients with invasive MRSA infection on admission to the intensive care unit, suspicion COVID-19, patients under the age of 18, and pregnant women were excluded from the study. Patients who were colonized with MRSA but died/discharged within the first 7 days of hospitalization were excluded from the study because analysis could not be performed.

### 2.2. Infection Control Precautions

In accordance with our hospital infection prevention protocol, swabs are taken from patients admitted to the intensive care unit during admission for MRSA screening. Patients who are confirmed to be MRSA carriers are placed in contact isolation. Contact isolation requires hand hygiene, wearing a gown and gloves.

### 2.3. Ethical Approval

This study was approved by local ethical committee (No:298 Date: 28 January 2025).

### 2.4. Microbiological Analysis

The swab samples taken from the patients were inoculated on blood agar (Salubris, İstanbul, Türkiye) medium and incubated at 35.5 °C to 37 °C for 24 to 48 h. The VITEK 2 system (Compact, bioMérieux, Marcy l'Etoile, France) was used to identify colonies growing on the medium. Antibiotic susceptibility testing was performed using the VITEK 2 system and Kirby–Bauer disk diffusion. The results were evaluated according to the recommendations of the European Committee on Antimicrobial Susceptibility Testing.

Weekly internal quality control strains are used for VITEK 2 Systems. Quality control tests for *S. aureus* are performed using the automated and standard Kirby–Bauer disk diffusion susceptibility test and the ETEST method (Bioanalyse, Ankara, Türkiye) with the ATCC 2913 Methicillin-Sensitive *S. aureus* strain.

### 2.5. Statistical Analysis

SPSS 22.0 (IBM Corp., Armonk, NY, USA) package program was used for statistical analysis. Categorical variables were expressed as numbers and percentages, and Chi-square was used for comparisons. For nonparametric data, Mann–Whitney U test was used between groups. Data that are not normally distributed will be displayed as median (min-max). In all analyses, *p*-value (two-tailed) < 0.05 will be considered statistically significant. Values with *p* < 0.05 were taken in binary logistic regression analysis.

## 3. Results

**Figure 1:**
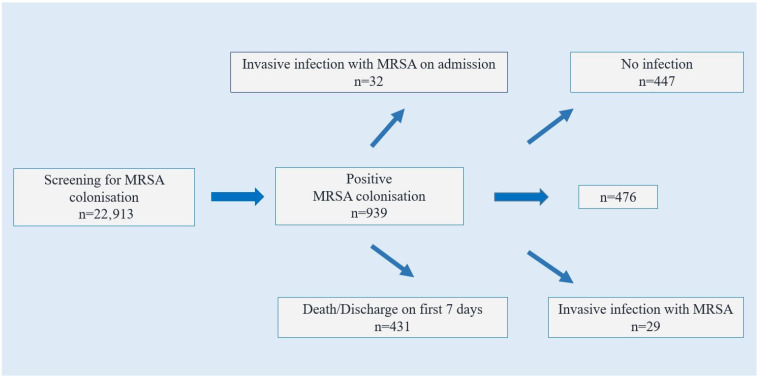
Flowchart of patients included in the study.

A total of 22,913 patients were tested with nasal swabs, and MRSA was isolated in 939 (4.0%) of them. Thirty-two of these patients had invasive MRSA infection and concurrent clinical culture growth on admission to intensive care unit. In addition, 431 patients who were discharged/exitus within the first seven days of hospitalization were excluded from the analysis. Of the 476 patients analysed, 29 (6.0%) developed invasive infections (Group 1), while 447 (94.0%) did not develop invasive infections (Group 2) within 28 days (Figure 1).

The demographic data and invasive procedures of the patients are presented in Table 1. According to this, there was no statistically significant difference between group 1 and 2 in terms of age and gender. Chronic kidney disease was present in 41.4% (*n* = 12) of group 1 and 14.3% (*n* = 64) of group 2 (*p* < 0.001). The proportion of patients undergoing routine hemodialysis was 20.7% (*n* = 6) in group 1 and 2.2% (*n* = 10) in group 2 (*p* < 0.001).

Central venous catheters were used in 96.6% (*n* = 28) of group 1 and 80.1% (*n* = 358) of group 2. The use of intubation, urinary catheterization, surgery, tracheostomy and percutaneous endoscopic gastrostomy was statistically similar between the groups (*p* > 0.05).

The rate of staying in a nursing home was 31.0% (*n* = 9) in group 1 and 6.5% (*n* = 29) in group 2 (*p* < 0.001). The rate of hospitalization in the last 90 days was 48.3% (*n* = 14) in group 1 and 27.3% (*n* = 122) in group 2 (*p* = 0.015).

According to multivariable regression analysis, routine hemodialysis (OR: 5.216, *p* = 0.015), staying in a nursing home (OR: 3.668, *p* = 0.014), and a history of hospitalization in the last 90 days (OR: 2.458, *p* = 0.028) were found to be risk factors for the development of invasive infection in MRSA carriers.

**Figure 2:**
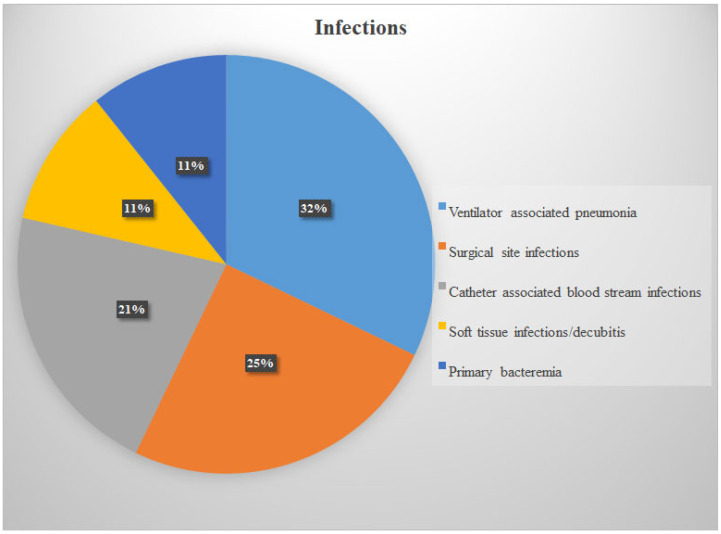
Infection types.

The most common invasive infections were ventilator-associated pneumonia (*n* = 9), surgical site infection (*n* = 7), and catheter-related bloodstream infection (*n* = 6) (Figure 2). All 29 strains were susceptible to vancomycin, linezolid, and daptomycin, while one strain was resistant to teicoplanin (3.5%). Resistance to quinolones was detected in 11 strains (37.9%), to clindamycin in 8 strains (27.5%), and to aminoglycosides in 6 strains (20.6%).

## 4. Discussion

In this retrospective study, the prevalence of nasal MRSA colonization among patients admitted to our adult intensive care unit was found to be 4.0%. Among patients with nasal MRSA colonization who met the follow-up criteria, 6.0% developed an invasive MRSA infection during their hospital stay. Multivariable analysis demonstrated that regular hemodialysis, a history of nursing home residence, and a history of hospitalization within the last 90 days were independent risk factors for the development of invasive infection in patients with MRSA colonization.

Although there are a large number of studies on MRSA prevalence, the target groups vary, including healthy peoples, healthcare workers, or patients [12,13,14,15]. MRSA prevalence in our region was reported as 1.2% in a study conducted approximately 10 years ago in the pulmonary diseases department [16]. A recent study found a prevalence of 11.3% among healthy students in Türkiye [17]. However, a meta-analysis identified a carrier rate of 10–20% in our region [18]. In a study from Türkiye, 900 patients were screened and MRSA was isolated in 11 (1.2%) patients. Multivariate analysis showed that insulin use was the only significant risk factor for MRSA colonization [19]. In our study, nasal MRSA colonization screening was performed in patients admitted to the intensive care unit to evaluate the prevalence of progression to invasive infection and risk factors. Our study has the largest patient population and the longest screening period conducted in our region and country to date. In our study, a prevalence of 4% was detected, and invasive infection was identified in 6% of colonized cases. Considering these relatively low rates, screening only patients with risk factors may be an approach for developing countries, considering staff, workload, and testing costs. Surveillance with screening cultures should be evaluated for cost-effectiveness for every patient admitted to the intensive care unit or hospital in developing countries. Conducting studies that include cost analyses on this matter will enable us to provide clearer explanations in the future.

In patients colonized with MRSA, the most important risk factor for developing invasive infection was routine hemodialysis in this study. The literature reports that MRSA colonization is significantly higher in hemodialysis patients compared to the general population. In a prospective study monitoring *S. aureus* colonization in hemodialysis patients, the *S. aureus* colonization rate varied cross-sectionally between 33% and 46% depending on the month and was found to be higher than in the general population [20]. In a study conducted by Sathish and colleagues, MRSA nasal colonization was found to be an independent risk factor for the development of MRSA bloodstream infections [21]. In a meta-analysis, the likelihood of progressing to an MRSA infection was 19% in colonized hemodialysis patients over the long term (6–20 months), compared to only 2% in non-colonized patients [22]. These data support the view that colonization and invasive infection are more common in this group. However, we were unable to obtain data showing how many patients had HD catheters or how many patients received HD treatment via a fistula. This data should be recorded in future studies.

The rate of nasal MRSA colonization of elderly patients in a nursing home in Brazil was 26% [23]. In Slovakia, it was found that staying in a geriatric care center nursing home and older age could be risk factors for the development of high-risk MRSA strains [24]. Recent hospitalization is also a risk factor for MRSA colonization [15,25]. In our study, both a history of nursing home stay and a history of hospitalization within the last 90 days were risk factors for the development of invasive MRSA infection from colonization. Since retrospective screening could not be performed prior to admission for these patients, the duration of colonization is unknown. Due to the retrospective nature of the study, no geriatric assessment or frailty scale was applied, and the pre-admission colonization period is unknown. Factors such as the immune system and colonization duration may have facilitated progression to invasive infection.

Intubation, urinary catheterization, and surgery did not show a statistically significant difference between the two groups. This may be due to the fact that these invasive procedures are performed on most patients in the intensive care unit. Studies conducted on a non-ICU, more general patient population may reach different conclusions on this matter.

## 5. Limitations

This study has some limitations. First, since this study is retrospective and single-center, the data may be limited. Second, MRSA colonization was detected only through nasal swabs; other colonization sites such as the perineum or throat were not screened, which may have led to an underestimation of the true prevalence. Another limitation is that, since only 29 patients developed invasive infections, it could lead to overfitting, leading to errors in the multivariable statistical model. Nearly half of MRSA-positive patients were excluded due to discharge or death within 7 days. On the other hand, infections that may have progressed to infection after discharge could not be followed up. Infections that developed after discharge may have been missed.

## 6. Conclusions

Our study has revealed the prevalence of MRSA colonization in patients admitted to the intensive care unit and the risk factors for progression to invasive infection. Based on our results, it should be discussed whether screening should be performed on selected patients rather than on all patients admitted to the ICU. This discussion must also include economic data. Hospitalization within the last 90 days, nursing home stay, and routine hemodialysis treatment are risk factors. Particularly in developing countries, rather than admitting all patients to the intensive care unit, considering the workload and costs, screening these three patient groups and implementing decolonization should be considered.

## Figures and Tables

**Table 1:** Features and risk factors of patients.

	Group 1 *n* = 29 (%)	Group 2 *n* = 447 (%)	Total *n* = 476 (%)	*p*	Odds Ratio (95% CI), *p*
Demographics					
Age	71 (18–99)	74 (18–108)	74 (18–108)	0.762	
Gender/Male	16 (55.0)	229 (51.0)	245 (51.0)	0.681	
Comorbidities					
Hypertension	10 (34.5)	132 (29.5)	142 (29.8)	0.572	
Coronary Artery Disease	14 (48.3)	173 (38.7)	187 (39.3)	0.306	
Coronary Artery Disease	1 (3.4)	66 (14.8)	67 (14.1)	0.089	
Chronic Obstructive Pulmonary Disease	4 (13.8)	77 (17.2)	81 (17.0)	0.634	
Congestive Heart Failure	4 (13.8)	67 (15.0)	71 (14.9)	0.861	
Chronic Kidney Disease	12 (41.4)	64 (14.3)	76 (16.0)	<0.001	1.333 (0.465–3.822), 0.593
Cancer	3 (10.3)	34 (7.6)	37 (7.8)	0.594	
Routine Hemodialysis	6 (20.7)	10 (2.2)	16 (3.4)	<0.001	5.216 (1.382–19.677), 0.015
Invasive Procedures					
Central venous catheter	28 (96.6)	358 (80.1)	386 (81.1)	0.028	5.312 (0.699–40.359), 0.107
Intubation	20 (69.0)	248 (55.5)	268 (56.3)	0.156	
Urinary catheter	28 (96.6)	402 (89.9)	430 (90.3)	0.242	
Surgical procedures	8 (27.6)	93 (20.8)	101 (21.1)	0.387	
Tracheostomy	4 (13.8)	35 (7.8)	39 (8.2)	0.257	
Percutaneous endoscopic gastrostomy	4 (14.3)	59 (13.2)	63 (13.3)	0.869	
Other					
Nursing home	9 (31.0)	29 (6.5)	38 (8.0)	<0.001	3.668 (1.300–10.351), 0.014
History of hospitalization in the last 90 days	14 (48.3)	122 (27.3)	136 (28.6)	0.015	2.458 (1.099–5.496), 0.028
Elective post-operative patients	7 (24.1)	101 (22.6)	108 (22.7)	0.848	

## Data Availability

The data supporting the findings of this study are not publicly available due to sensitivity and are available from the corresponding author upon reasonable request.
